# AAV9-mediated transduction of memory circuits following convection-enhanced delivery into the olfactory bulbs

**DOI:** 10.1038/s41434-025-00555-4

**Published:** 2025-07-26

**Authors:** Theodore Dimitrov, Vikas Munjal, Allison O’Brien, Matthew T. Rocco, Ahmad Karkhah, Kaya E. Ceyhan, Daniel Prevedello, Lluis Samaranch

**Affiliations:** 1 Department of Neurological Surgery, The Ohio State University, Columbus, OH USA; 2 Gene Therapy Institute, The Ohio State University, Columbus, OH USA; 3 Medical Student Research Program, The Ohio State College of Medicine, The Ohio State University Wexner Medical Center, Columbus, OH USA

**Keywords:** Olfactory system, Cellular neuroscience

## Abstract

This study explores the potential of adeno-associated virus serotype 9 (AAV9) to deliver therapeutic genes directly into the memory circuit throughout the olfactory bulb (OB), a critical memory and sensory processing region. Using convection-enhanced delivery (CED) of AAV9 encoding green fluorescent protein (GFP), we mapped the extensive neural connectivity from the OB to key memory-related brain regions, including the entorhinal cortex (EC) and hippocampus. Our findings reveal significant transduction of neural pathways and underscore the potential of targeting the OB connectome for therapeutic interventions in progressive neurodegenerative disorders such as Alzheimer’s disease or mild cognitive impairment. Targeting the OB connectome will pave the way for new therapeutic strategies to preserve neuronal function and slow the progression, offering a promising avenue beyond symptomatic relief to address the underlying mechanisms of the disease.

## Introduction

The olfactory system is a unique sensory pathway that exerts widespread influence on cognitive and emotional functions [1–5]. Rhythmic oscillations of the olfactory bulbs (OBs), synchronized with respiration, play a critical role in modulating sensory processing, neural synchrony, and interregional communication across the brain [1, 4, 6, 7]. These oscillations, particularly in the theta (4–8 Hz) and gamma (30–100 Hz) frequency bands, are tightly coupled with memory formation, learning, and emotional regulation [8]. The OBs are functionally connected to key cortical memory structures, including the entorhinal cortex (EC) and hippocampus, and disruptions in OB-EC-hippocampus synchrony have been shown to impair recognition memory and precede structural degeneration in preclinical models [9, 10].

This functional network is particularly relevant in the context of neurodegenerative diseases such as Alzheimer’s disease (AD), which is characterized by early pathological changes in the EC [11–13] AD pathophysiology—including beta-amyloid plaque deposition, neurofibrillary tangles, and synaptic loss—follows patterns that support the network degeneration hypothesis, suggesting selective vulnerability of highly interconnected brain regions. Importantly, olfactory dysfunction often manifests before overt cognitive decline, and AD pathology has been detected in the olfactory epithelium and OBs during early disease stages [14–23]. These findings position the olfactory system as a promising biomarker and a potential entry point for early therapeutic intervention in AD-related neurodegeneration.

In this study, we demonstrate the therapeutic potential of direct OB delivery of AAV9 vectors. By utilizing AAV9 vectors to deliver therapeutic agents directly to the OB, we confirmed its unique position within the brain’s circuitry to achieve broad transgene expression in memory circuits and other related regions. While AD serves as a primary example of the relevance of this approach, the implications extend to other neurodegenerative diseases and conditions characterized by disruptions in interregional connectivity. The unique position of the olfactory system at the intersection of sensory processing, memory formation, and emotional regulation highlights its potential as a gateway for innovative therapies that address the complex interplay of factors underlying cognitive and emotional dysfunction.

## Material and methods

### Viral vector and experimental design

Four male C57BL/6 WT mice ( ~ 21–28 g) received a unilateral injection of an AAV9 vector encoding the GFP transgene (AAV9-GFP) into the right olfactory bulb utilizing convection-enhanced delivery. Briefly, animals were anesthetized with isoflurane (Baxter, Deerfield, USA), administered a subcutaneous injection of NSAID (Meloxicam) and buprenorphine (Buprenorphine ER-Lab), and placed in a stereotactic frame. A longitudinal incision was made in the skin overlying the skull, and a burr-hole was drilled according to olfactory bulb stereotactic coordinates (AP: 5.2, ML: -1.0, DV: -2.5). A custom-made fused silica cannula with a 0.5-mm step was used to infuse 3 μL of the vector into the right OB at a rate of 1 µL/min. Once infusion was completed, the skin was sutured, and a topical antibiotic was applied to the incision line. Animals were removed from the stereotaxic frame and monitored until recovered. Then, they returned to their cages and were evaluated twice daily for five days. Animals were kept in a 12:12 h light/dark cycle, and the temperature and humidity of the animal room was maintained at 21 ± 2 °C and 50–60%, respectively. Further, animals received Motrin water for 72 h post operatively. All animals had free access to food and water.

The viral vector (AAV9-CBA-eGFP) was manufactured at the Penn Vector Core (University of Pennsylvania) at a titer of 8.95E + 13 GC/mL. All the experiments were performed in accordance with the OSU Institutional Animal Care and Use Committee.

### Tissue processing

Animals were followed for 3 weeks. On the day of sacrifice, animals were euthanized with carbon dioxide inhalation and then promptly transcardially perfused with PBS, followed by 4% paraformaldehyde (PFA)/PBS. Brains were harvested, post-fixed in 4% PFA/PBS for 24–48 h, and cryoprotected in 30% sucrose. All post-fixed brains were cut into 40 μm serial coronal sections and processed for immunofluorescence staining against GFP and NeuN.

### Immunofluorescent staining

Double immunolabeling was performed to assess vector distribution and GFP signal transduction against background neuronal cell labeling with NeuN nuclear protein for anatomical reference. Briefly, representative brain sections were washed with PBST, blocked for 30 min in 1X Animal Free Blocker (AFB, Vector Laboratories), and incubated with rabbit anti-GFP (1:1000, Ref: 610362 Invitrogen) and chicken anti-NeuN (1:1000, Ref: GTX00837 GenTex) overnight at 4°C. The next day, sections were washed in PBST and incubated with a cocktail of secondary antibodies (Donkey anti-rabbit, 1:500, Ref: A32790 Invitrogen; Goat anti-chicken, 1:500, Ref: A32932 Invitrogen) in 1X AFB for 1 h at room temperature, washed with PBS and wet mounted.

## Results and discussion

Three weeks after convection-enhanced delivery into the olfactory bulbs of wild-type mice of 3 µl of AAV9 encoding GFP under the control of a chicken-β-actin (CBA) promoter at 2.7E + 11 total GC, we observed robust GFP expression across distinct projection sites, indicating successful transduction and transport through interconnected circuits (Supplementary Fig. 1).

Our study reveals the comprehensive map of the neural pathways originating from the OB and their extensive connectivity with critical brain regions involved in memory and sensory processing. We visualized and confirmed several intricate pathways by employing GFP transduction via the AAV9 viral vector, elucidating the functional relationships between the OB and various memory-related downstream targets, including the hippocampus and EC. These findings provide a crucial understanding of the olfactory-hippocampal circuitry and its potential implications for drug delivery in neurodegenerative diseases, particularly AD.

**Fig. 1:**
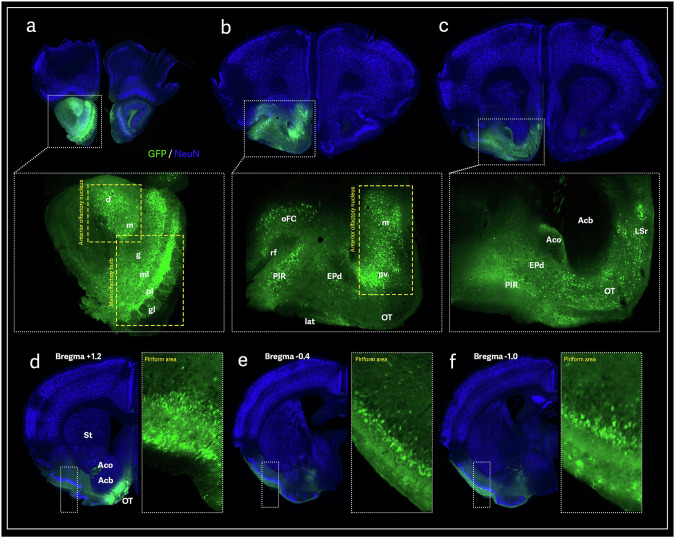
AAV9-mediated GFP transduction and distribution in the anterior mouse olfactory-associated circuitry. A representative brain section shows transduction in the olfactory bulb at the prefrontal cortex following AAV9-GFP olfactory tract injection **a**. GFP transduction was traced through the piriform area, revealing GFP-positive cells in the orbitofrontal cortex, endopiriform nucleus, and anterior olfactory nucleus **b**. The GFP-positive signal was also observed along the olfactory tracts and the septal nucleus **c**, as well as along the anterior-posterior axis of the piriform area **d–f**. Anterior olfactory nucleus, dorsal part (d); Anterior olfactory nucleus, medial part (m); Main olfactory bulb, granular layer (g); Main olfactory bulb, mitral layer (ml); Main olfactory bulb, plexiform layer (pl); Main olfactory bulb, glomerular layer (gl); Orbital cortex area, frontal (oFC); Rhinal fissure (rf); Piriform area, molecular layer (PIR); Lateral olfactory tract (lat); Endopiriform nucleus, dorsal part (EPd); Anterior olfactory nucleus, posteroventral part (pv); Olfactory tubercle (OT); Lateral septal nucleus, rostral part (LSr); Anterior commissure, olfactory limb (Aco); Nucleus accumbens (Acb).

**Fig. 2:**
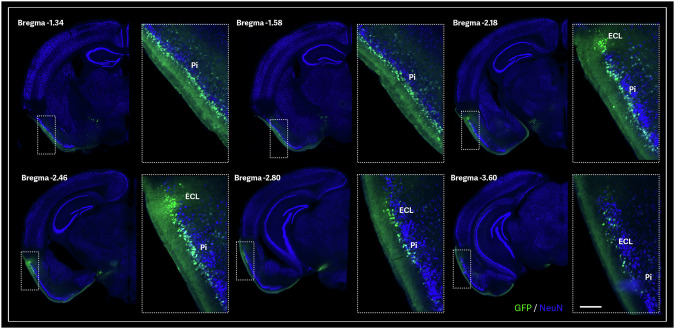
AAV9-mediated axonal transport in the mouse retrohippocampal region. Representative brain sections show a GFP-positive signal along the anterior-posterior axis following AAV9-GFP injection into the olfactory bulb. Piriform area, molecular layer (Pi); Entorhinal area, lateral part (ECL). Scale bar: 200 µm.

The AAV9-GFP infusion into the OB resulted in strong local transduction, including the granular, mitral, plexiform, and glomerular layers (Fig. 1a). In the OB, olfactory sensory neurons project their axons to the anterior olfactory nucleus (AON) throughout the olfactory tracts in the olfactory peduncle [24]. The AON is a region involved in the central processing of olfactory information. Its connections with the hippocampus modulate episodic, spatial, and temporal odor memory [25]. GFP-positive cells were found in the AON, especially in the dorsal and medial portions (Fig. 1b). From AON, the pathway continues to the piriform area (PIR), the largest component of the olfactory cortex [25]. The PIR has been described as a critical area for odor encoding and memory [25, 26]. GFP transduction followed along the piriform area, showing GFP-positive cells in the orbitofrontal cortex and endopiriform nucleus (Fig. 1b, c). Within the PIR extending posteriorly (Fig. 1d–f), the GFP signal extended to the lateral and medial entorhinal cortices (LEC and MEC), critical relay stations to the hippocampus (Fig. 2). The MEC processes general spatial information and interacts with the hippocampus to support spatial memory and navigation through grid cells [27].

**Fig. 3:**
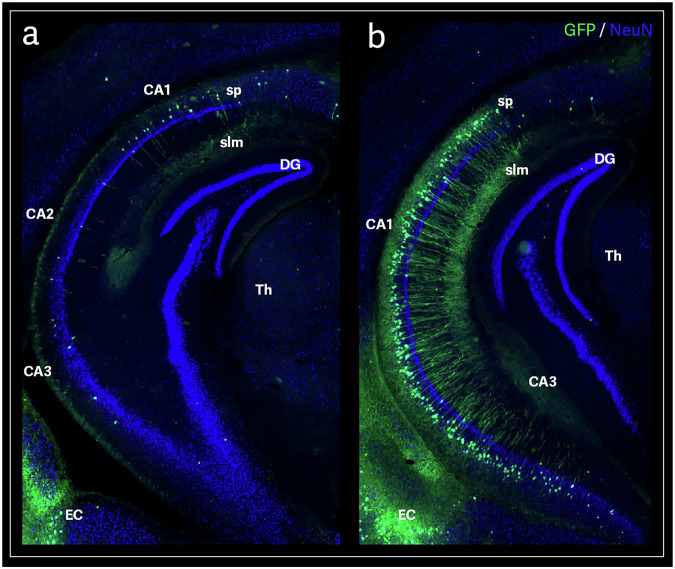
AAV9-mediated axonal transport in the mouse hippocampal region. A representative anterior brain section showing GFP-positive signal after AAV9-GFP olfactory-bulb injection **a**. A representative posterior brain section showing a strong CA1 neuronal GFP transduction after AAV9-GFP olfactory-bulb injection **b**. Field CA1 (CA1); Field CA2 (CA2); Field CA3 (CA3); CA1 field, pyramidal layer (sp); CA1 field, stratum lacunosum-moleculare (slm); Dentate gyrus, granular cell layer (DG); Thalamus (Th); Entorhinal area (EC).

In contrast, the lateral entorhinal cortex (LEC) is crucial for encoding object-related information, including their locations, and works with the hippocampus to integrate these details into broader contextual memories [27]. Together, the MEC and LEC enable the hippocampus to form detailed, context-rich memories, combining spatial layouts with specific item-related data [27]. The LEC and MEC further transmitted the GFP signal to the dentate gyrus (DG) and hippocampal subfields CA3, CA1, and the subiculum, following the canonical trisynaptic circuit of the hippocampus (Fig. 3), demonstrating a direct pathway from the OB to the hippocampus.

**Fig. 4:**
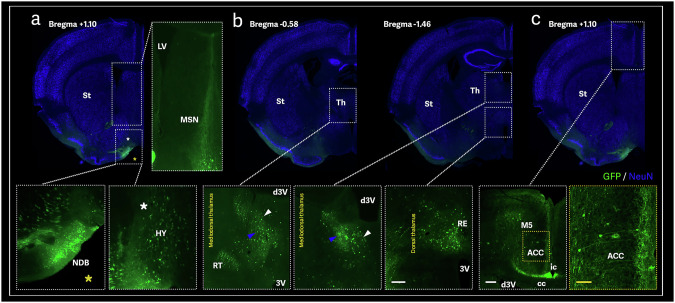
AAV9-mediated axonal transport in the mouse brain throughout the olfactory tubercle connectome. Representative sections showing GFP-positive signal at the medial septal complex **a**, thalamus **b** and cingulate **c** levels after AAV9-GFP olfactory-bulb injection. Striatum (st); Lateral ventricle (LV); Medial septal complex (MSN); Diagonal band nucleus (NDB); Hypothalamus (HY); Thalamus (Th); Dorsal 3^rd^ ventricle (d3V); 3^rd^ ventricle (3V); Medial mediodorsal thalamus (blue arrowhead); Lateral mediodorsal thalamus (white arrowhead); Reticular nucleus thalamus (RT); Nucleus of reuniens (RE); Anterior cingulate cortex (ACC); Corpus callosum (cc); Induseum griseum (ic); Motor area, layer 5 (M5) Piriform area, molecular layer (Pi); Entorhinal area, lateral part (ECL). Scale bar: 200 µm (white), 100 µm (yellow).

Another significant pathway transduced throughout OB infusion involved the olfactory tubercle (OT) at the base of the forebrain (Fig. 1c). GFP signal was observed in the medial septal complex (MSN + NDB) from the medial pallidum, continuing to the mediodorsal thalamus (MDT) (Figs. 4a, b). The thalamic projections then can be seen relaying the signal to the anterior cingulate cortex (ACC) and *induseum griseum* (Fig. 4c), a remnant of the former position of the hippocampus connecting to the ventral hippocampus (vHPC) to mediate emotion and reward-related processing [28]. The ACC, an anatomically distinct sub-region of the ventromedial prefrontal cortex (PFC), is crucial for various aspects of cognition, including attention, cognitive control, working memory, set maintenance, and goal-directed behavior [29, 30]. Higher gray matter volumes in the ACC have been linked to the cognitive flexibility component of executive functions, as evidenced by performance on measures of task switching [30, 31]. Although data is limited, the ACC’s involvement in multiple cognitive processes, particularly executive function, has been described [32, 33]. The loss of ACC volume has been observed in patients with amnestic mild cognitive impairment (aMCI) who progressed to AD [33]. Functional MRI (fMRI) studies have also shown degeneration and changes in functional activity in the ACC of patients with early AD, indicating its vulnerability and critical role in the disease’s progression [32, 33].

**Fig. 5:**
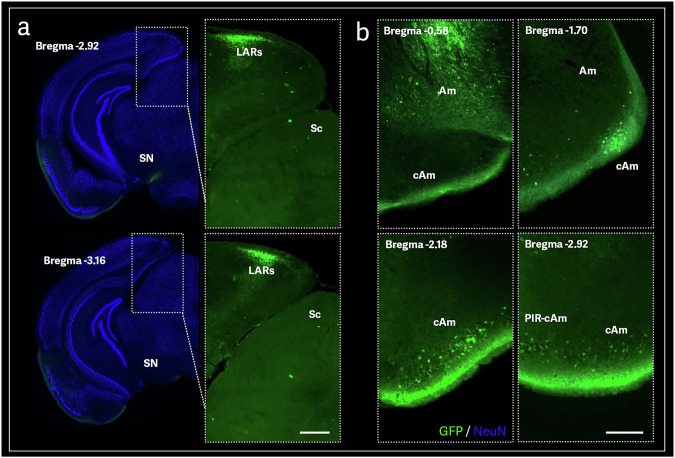
AAV9-mediated axonal transport in the mouse brain throughout limbic system components. Representative sections showing GFP-immunoreactive cells at the rodent equivalent of the primate cingulate **a** and amygdala **b** after AAV9-GFP olfactory-bulb injection. Retrosplenial area, lateral agranular part (LARs); Superior colliculus (Sc); Substantia nigra (SN); Amygdalar cortex (cAM); Amygdalar area (Am); Piriform-amygdalar area (PIR-cAm). Scale bar: 200 µm.

The ACC has further connections to the posterior cingulate gyrus (PCC), a critical component of the default mode network [34]. PCC plays a significant role in conditions such as autism, depression, and attention-deficit/hyperactivity disorder but is particularly vulnerable in the early AD stages [34]. Still, the PCC-synchronized degeneration network (PCC-SDN), rather than the hippocampus-SDN, has been more closely associated with AD progression [35]. The PCC-SDN involves widespread frontal, temporal, insular, and cerebellar areas, highlighting its versatility in integrating and modulating multimodal sensory information [35]. This network supports spatial processing, episodic memory, self-reflection, attention, value assessment, and various adaptive behaviors [35, 36]. Recent research by Xiang et al. 2023 identified the location and extent of a possible rodent equivalent of the primate PCC, the lateral agranular retrosplenial cortex (LARs) [36]. Our study found GFP-positive staining in this location after the OB injection, providing further insight into the LARs’ role in the rodent olfactory pathway and its potential relevance to AD disease therapy (Fig. 5a). These networks emphasize the role of the olfactory system in broader cognitive and emotional processing. The integration of olfactory information into these regions and their subsequent relay to the PFC suggests a complex interplay between olfaction, executive functions, and memory. The emotional processing network, closely intertwined with memory pathways, involves structures such as the vHPC, amygdala, and the orbitofrontal cortex. This network facilitates encoding and retrieving emotional memories, modulating responses to emotional stimuli, and regulating mood and affect [37]. GFP immunoreactive cells and fibers were found in these structures (Figs. 1b, 3b, 5b).

Additionally, the OT projects to the PIR, following the same route through the EC to the hippocampus as in the primary pathway [38, 39]. This suggests a multifaceted role of olfactory pathways in linking sensory perception with cognitive and emotional dimensions of brain function.

A secondary anatomical pathway connects the OB with the cortical amygdalar area (cAm) and the basolateral amygdala (BLA) throughout the olfactory tracts and PIR [40]. The lateral nucleus of BLA receives sensory input from the thalamus and the cortex [40]. GFP-positive staining was found in these areas, especially in the BLA, which includes the lateral and basal nuclei (Fig. 5b). The cortical amygdala is primarily involved in olfactory processing, contributing to emotional responses triggered by smells [25]. Conversely, the BLA is crucial for emotional learning and memory, associating sensory experiences with emotional significance [25]. The BLA then projects to the central amygdala (CeA), a key regulator of emotional processing and response, which transmits signals to the lateral hypothalamus (LH) [41]. GFP positive signal was also found along this pathway (Fig. 4a). As the PIR, the amygdala-centered pathway also engaged the MEC, projecting to the hippocampal DG, CA3, CA1, and subiculum.

**Fig. 6:**
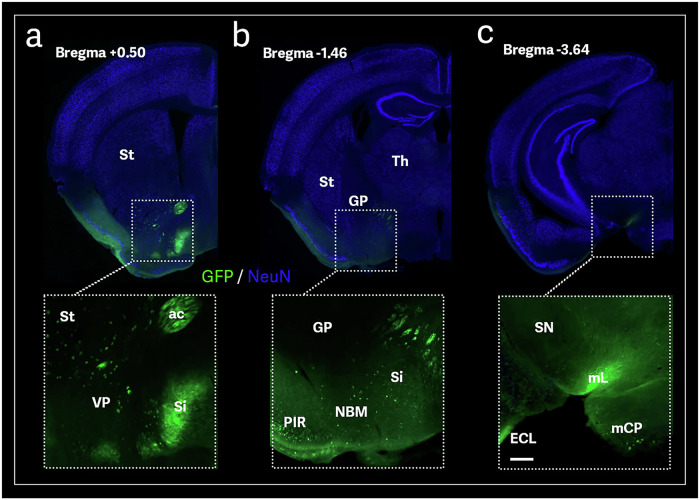
AAV9-mediated axonal transport in the mouse brain through memory, learning, and motor structures after AAV9-GFP olfactory-bulb injection. Representative sections showing GFP staining in different components of the dorsal cholinergic system **a**, **b** and sensorimotor functions **c**. Striatum (st); anterior commissure (ac); Substantia innominate (si); Ventral pallidum (VP); Globus pallidus (GP); Nucleus basalis of Meyner (NBM); Piriform area, molecular layer (PIR); Substantia nigra (SN); Medial laminiscus (mL); Middle cerebellar peduncle (mCP); Entorhinal area, lateral part (ECL). Scale bar: 200 µm.

Additionally, GFP-immunoreactive cells were observed in the *substantia innominata* (SI), including the nucleus basalis of Meynert (NBM) (Fig. 6a and 6b). These structures provide rich cholinergic projections to the neocortex, an essential structure for learning and memory [42]. They also project to the hippocampus, influencing synaptic plasticity and long-term potentiation, which is also necessary for memory encoding and consolidation [42]. Degeneration in the cholinergic system within the SI, especially in the NBM, is associated with AD and other degenerative diseases [43]. Recent models of AD suggest that degeneration of cholinergic neurons in the NBM precedes neuropathological changes in the medial temporal lobe, specifically the entorhinal cortex [43]. This loss of cholinergic input from the NBM to the cortex and hippocampus correlates with the severity of cognitive decline in AD patients [43].

We also found GFP positive signal in the medial lemniscus and middle cerebellar peduncle (Fig. 6c), highlighting the integration of olfactory information with other sensory modalities like sensorimotor functions and environmental responsiveness [44]. Typically, patients with AD experience a decline in motor coordination, balance, and daily activities, beginning in the early stages and progressing until they become entirely dependent on others for all aspects of daily living in the more advanced stages [45].

These findings extend a growing body of literature that highlights the olfactory system’s central role in modulating cognition, emotion, and interregional communication [1, 4]. By tracing AAV9-GFP-labeled pathways from the OB to a wide range of downstream targets—including the piriform cortex, entorhinal cortex, hippocampus, anterior and posterior cingulate cortices, amygdala, basal forebrain, and even cerebellar-associated tracts—we provide anatomical confirmation of the OB’s extensive influence on distributed brain networks critical for memory formation, emotional regulation, and executive function. The identification of projections through the medial entorhinal cortex (MEC) and lateral entorhinal cortex (LEC) to the hippocampus reinforces prior findings that neonatal olfactory activity is necessary for the structural and functional development of hippocampal memory circuits [3, 5]. Moreover, the engagement of canonical hippocampal subfields (DG, CA3, CA1, and subiculum) following OB transduction suggests that the olfactory system plays a sustained role in supporting memory retrieval and spatial encoding beyond early development [35, 36].

The widespread anatomical projections revealed in this study also support the hypothesis that the OB helps synchronize activity across distant brain regions. Prior studies have established that rhythmic OB oscillations, tightly coupled to respiration, can entrain theta and gamma activity in areas such as the EC and hippocampus—frequencies that are vital for episodic memory and cognitive flexibility [6, 9, 10]. Our observed projections from the OB to the ACC, PCC, and lateral agranular retrosplenial cortex (LARs) provide additional structural pathways by which olfactory input may influence the broader default mode network, a system known to be disrupted in the earliest stages of Alzheimer’s disease (AD) [16, 28–36, 46]. The identification of OB projections to the substantia innominata and nucleus basalis of Meynert further suggests a role in neuromodulatory regulation via cholinergic pathways—another circuit that degenerates early in AD [42]. These anatomical routes offer potential mechanisms for how olfactory system dysfunction could contribute not only to early sensory deficits, but also to broader impairments in memory, attention, and affect regulation.

Importantly, our findings support the network degeneration hypothesis of AD, which posits that disease pathology propagates through functionally and anatomically connected brain regions rather than emerging randomly [14, 16–19]. The structural connections we identified from the OB to regions preferentially affected in early AD—such as the EC, hippocampus, PCC, and basal forebrain—mirror the networks shown to degenerate first in both clinical and preclinical models. The presence of GFP-labeled pathways to the basolateral amygdala, cortical amygdalar area, and ventral hippocampus underscores the OB’s involvement in emotional memory circuits, while projections to the cerebellar peduncle and medial lemniscus suggest its role in integrating sensory and motor processes—domains that are increasingly recognized as vulnerable in neurodegenerative disease.

This is particularly relevant in the context of neurodegenerative diseases like Alzheimer’s, where the earliest pathological changes frequently appear in olfactory regions before clinical symptoms emerge. The observation that olfactory dysfunction often precedes measurable cognitive decline has raised interest in the OB as both a diagnostic and therapeutic target. Our study’s findings directly support this notion by tracing the OB’s connectivity to vulnerable cortical and limbic areas, many of which are among the first affected in Alzheimer’s disease. Furthermore, the olfactory system’s accessibility via the nasal cavity opens promising avenues for non-invasive, targeted delivery of therapeutic agents to memory-related regions. By demonstrating the structural routes through which such agents could potentially reach areas like the hippocampus, amygdala, and prefrontal cortex, our results provide a crucial anatomical framework for advancing these strategies.

Taken together, this work contributes to a deeper understanding of how olfactory inputs influence broader cognitive and emotional circuits, reinforces the OB’s role in organizing memory-relevant activity, and highlights the potential of olfactory pathways as a conduit for early intervention in neurodegenerative disease.

## Supplementary information

Supplementary Fig.1.

## References

[1] Bagur S, Lefort JM, Lacroix MM, de Lavilléon G, Herry C, Chouvaeff M, et al. Breathing-driven prefrontal oscillations regulate maintenance of conditioned-fear evoked freezing independently of initiation. Nat Commun. 2021;12:2605. 33972521 10.1038/s41467-021-22798-6 PMC8110519

[2] Chen Y-N, Kostka JK, Bitzenhofer SH, Hanganu-Opatz IL. Olfactory bulb activity shapes the development of entorhinal-hippocampal coupling and associated cognitive abilities. Curr Biol. 2023;33:4353–66.e5. 37729915 10.1016/j.cub.2023.08.072 PMC10617757

[3] Kostka JK, Hanganu-Opatz IL. Olfactory-driven beta band entrainment of limbic circuitry during neonatal development. J Physiol. 2023;601:3605–30. 37434507 10.1113/JP284401

[4] Moberly AH, Schreck M, Bhattarai JP, Zweifel LS, Luo W, Ma M. Olfactory inputs modulate respiration-related rhythmic activity in the prefrontal cortex and freezing behavior. Nat Commun. 2018;9:1528. 29670106 10.1038/s41467-018-03988-1 PMC5906445

[5] Yahiaoui-Doktor M, Luck T, Riedel-Heller SG, Loeffler M, Wirkner K, Engel C. Olfactory function is associated with cognitive performance: results from the population-based LIFE-Adult-Study. Alzheimer’s Res Ther. 2019;11:43. 31077241 10.1186/s13195-019-0494-z PMC6511191

[6] Ghazvineh S, Salimi M, Nazari M, Garousi M, Tabasi F, Dehdar K, et al. Rhythmic air-puff into nasal cavity modulates activity across multiple brain areas: A non-invasive brain stimulation method to reduce ventilator-induced memory impairment. Respir Physiol Neurobiol. 2021;287:103627. 33516946 10.1016/j.resp.2021.103627

[7] Salimi M, Ghazvineh S, Zare M, Parsazadegan T, Dehdar K, Nazari M, et al. Distraction of olfactory bulb-medial prefrontal cortex circuit may induce anxiety-like behavior in allergic rhinitis. PLoS ONE. 2019;14:e0221978. 31509547 10.1371/journal.pone.0221978 PMC6738655

[8] Lisman JE, Jensen O. The θ-γ neural code. Neuron. 2013;77:1002–16. 23522038 10.1016/j.neuron.2013.03.007 PMC3648857

[9] Salimi M, Tabasi F, Abdolsamadi M, Dehghan S, Dehdar K, Nazari M, et al. Disrupted connectivity in the olfactory bulb-entorhinal cortex-dorsal hippocampus circuit is associated with recognition memory deficit in Alzheimer’s disease model. Sci Rep. 2022;12:4394. 35292712 10.1038/s41598-022-08528-y PMC8924156

[10] Khan UA, Liu L, Provenzano FA, Berman DE, Profaci CP, Sloan R, et al. Molecular drivers and cortical spread of lateral entorhinal cortex dysfunction in preclinical Alzheimer’s disease. Nat Neurosci. 2014;17:304–11. 24362760 10.1038/nn.3606 PMC4044925

[11] Li X, Feng X, Sun X, Hou N, Han F, Liu Y. Global, regional, and national burden of Alzheimer’s disease and other dementias, 1990–2019. Front Aging Neurosci. 2022;14:937486. 36299608 10.3389/fnagi.2022.937486 PMC9588915

[12] Gómez-Isla T, Price JL, McKeel JrDW, Morris JC, Growdon JH, Hyman BT. Profound loss of layer II entorhinal cortex neurons occurs in very mild Alzheimer’s disease. J Neurosci. 1996;16:4491–500. 8699259 10.1523/JNEUROSCI.16-14-04491.1996 PMC6578866

[13] Igarashi KM. Entorhinal cortex dysfunction in Alzheimer’s disease. Trends Neurosci. 2023;46:124–36. 10.1016/j.tins.2022.11.006 PMC9877178 36513524

[14] Chételat G. Multimodal neuroimaging in Alzheimer’s disease: early diagnosis, physiopathological mechanisms, and impact of lifestyle. J Alzheimer’s Dis. 2018;64:S199–211. 29504542 10.3233/JAD-179920 PMC6004909

[15] DeTure MA, Dickson DW. The neuropathological diagnosis of Alzheimer’s disease. Mol Neurodegenerat. 2019;14:32. 10.1186/s13024-019-0333-5 PMC6679484 31375134

[16] Greicius MD, Srivastava G, Reiss AL, Menon V. Default-mode network activity distinguishes Alzheimer’s disease from healthy aging: Evidence from functional MRI. Proc Natl Acad Sci. 2004;101:4637–42. 15070770 10.1073/pnas.0308627101 PMC384799

[17] Hari E, Kizilates-Evin G, Kurt E, Bayram A, Ulasoglu-Yildiz C, Gurvit H, et al. Functional and structural connectivity in the Papez circuit in different stages of Alzheimer’s disease. Clin Neurophysiol. 2023;153:33–45. 37451080 10.1016/j.clinph.2023.06.008

[18] Seeley WW, Crawford RK, Zhou J, Miller BL, Greicius MD. Neurodegenerative diseases target large-scale human brain networks. Neuron. 2009;62:42–52. 19376066 10.1016/j.neuron.2009.03.024 PMC2691647

[19] Zhou J, Gennatas Efstathios D, Kramer Joel H, Miller Bruce L, Seeley William W. Predicting Regional Neurodegeneration from the Healthy Brain Functional Connectome. Neuron. 2012;73:1216–27. 22445348 10.1016/j.neuron.2012.03.004 PMC3361461

[20] Son G, Jahanshahi A, Yoo SJ, Boonstra JT, Hopkins DA, Steinbusch HWM, et al. Olfactory neuropathology in Alzheimer’s disease: a sign of ongoing neurodegeneration. BMB Rep. 2021;54:295–304. 34162463 10.5483/BMBRep.2021.54.6.055 PMC8249876

[21] Winchester RL, Martyn K. Could Early Identification of Changes in Olfactory Function Be an Indicator of Preclinical Neurodegenerative Disease? A Systematic Review. Neurol Ther. 2020;9:243–63. 32529479 10.1007/s40120-020-00199-z PMC7606376

[22] Dintica CS, Marseglia A, Rizzuto D, Wang R, Seubert J, Arfanakis K, et al. Impaired olfaction is associated with cognitive decline and neurodegeneration in the brain. Neurology. 2019;92:e700–e9. 30651382 10.1212/WNL.0000000000006919 PMC6382360

[23] Talamo BR, Rudel R, Kosik KS, Lee VM, Neff S, Adelman L, et al. Pathological changes in olfactory neurons in patients with Alzheimer’s disease. Nature. 1989;337:736–9. 2465496 10.1038/337736a0

[24] Collins LN, Brunjes PC. The mouse olfactory peduncle 4: Development of synapses, perineuronal nets, and capillaries. J Comp Neurol. 2020;528:637–49. 31571216 10.1002/cne.24778 PMC6944759

[25] Zhang J, Zhao Z, Sun S, Li J, Wang Y, Dong J, et al. Olfactory evaluation in Alzheimer’s disease model mice. Brain Sci. 2022;12:607. 35624994 10.3390/brainsci12050607 PMC9139301

[26] Murphy C. Olfactory and other sensory impairments in Alzheimer disease. Nat Rev Neurol. 2019;15:11–24. 30532084 10.1038/s41582-018-0097-5

[27] Save E, Sargolini F. Disentangling the role of the MEC and LEC in the processing of spatial and non-spatial information: contribution of lesion studies. Front Syst Neurosci. 2017;11:81. 29163076 10.3389/fnsys.2017.00081 PMC5663729

[28] Rolls ET. The cingulate cortex and limbic systems for emotion, action, and memory. Brain Struct Funct. 2019;224:3001–18. 31451898 10.1007/s00429-019-01945-2 PMC6875144

[29] Barbey AK, Koenigs M, Grafman J. Orbitofrontal contributions to human working memory. Cereb Cortex. 2011;21:789–95. 20724371 10.1093/cercor/bhq153 PMC3059885

[30] Nee DE, Kastner S, Brown JW. Functional heterogeneity of conflict, error, task-switching, and unexpectedness effects within medial prefrontal cortex. Neuroimage. 2011;54:528–40. 20728547 10.1016/j.neuroimage.2010.08.027 PMC2962721

[31] Huster RJ, Wolters C, Wollbrink A, Schweiger E, Wittling W, Pantev C, et al. Effects of anterior cingulate fissurization on cognitive control during stroop interference. Hum Brain Mapp. 2009;30:1279–89. 18570202 10.1002/hbm.20594 PMC6870757

[32] Jung F, Kazemifar S, Bartha R, Rajakumar N. Semiautomated assessment of the anterior cingulate cortex in Alzheimer’s disease. J Neuroimaging. 2019;29:376–82. 30640412 10.1111/jon.12598

[33] Yuan Q, Liang X, Xue C, Qi W, Chen S, Song Y, et al. Altered anterior cingulate cortex subregional connectivity associated with cognitions for distinguishing the spectrum of pre-clinical Alzheimer’s disease. Front Aging Neurosci. 2022;14:1035746. 36570538 10.3389/fnagi.2022.1035746 PMC9768430

[34] Leech R, Sharp DJ. The role of the posterior cingulate cortex in cognition and disease. Brain. 2014;137:12–32. 23869106 10.1093/brain/awt162 PMC3891440

[35] Lee P-L, Chou K-H, Chung C-P, Lai T-H, Zhou JH, Wang P-N, et al. Posterior cingulate cortex network predicts Alzheimer’s disease progression. Front Aging Neurosci. 2020;12:608667. 33384594 10.3389/fnagi.2020.608667 PMC7770227

[36] Xiang X-J, Chen S-Q, Zhang X-Q, Chen C-H, Zhang S-Y, Cai H-R, et al. Possible rodent equivalent of the posterior cingulate cortex (area 23) interconnects with multimodal cortical and subcortical regions. Front Neurosci. 2023;17:1194299. 37383104 10.3389/fnins.2023.1194299 PMC10293749

[37] Torrico T, Abdijadid S. Neuroanatomy, Limbic System. StatPearls. StatPearls Publishing, Treasure Island; 2023. 30860726

[38] García-Cabezas MÁ, Barbas H. A direct anterior cingulate pathway to the primate primary olfactory cortex may control attention to olfaction. Brain Struct Funct. 2014;219:1735–54. 23797208 10.1007/s00429-013-0598-3 PMC5028194

[39] Wesson DW, Wilson DA. Sniffing out the contributions of the olfactory tubercle to the sense of smell: hedonics, sensory integration, and more?. Neurosci Biobehav Rev. 2011;35:655–68. 20800615 10.1016/j.neubiorev.2010.08.004 PMC3005978

[40] McDonald AJ. Functional neuroanatomy of the basolateral amygdala: Neurons, neurotransmitters, and circuits. Handbook of behavioral neuroscience. 26: Elsevier; 2020. p. 1-38. 10.1016/b978-0-12-815134-1.00001-5 PMC8248694 34220399

[41] Weera MM, Shackett RS, Kramer HM, Middleton JW, Gilpin NW. Central amygdala projections to lateral hypothalamus mediate avoidance behavior in rats. J Neurosci. 2021;41:61–72. 33188067 10.1523/JNEUROSCI.0236-20.2020 PMC7786206

[42] Ballinger EC, Ananth M, Talmage DA, Role LW. Basal forebrain cholinergic circuits and signaling in cognition and cognitive decline. Neuron. 2016;91:1199–218. 27657448 10.1016/j.neuron.2016.09.006 PMC5036520

[43] Mieling M, Meier H, Bunzeck N. Structural Degeneration of the Nucleus basalis of Meynert in Mild Cognitive Impairment and Alzheimer’s disease–evidence from an MRI-based meta-analysis. Neurosci Biobehav Rev. 2023;154:105393. 10.1016/j.neubiorev.2023.105393 37717861

[44] Liu X, Huang H, Snutch TP, Cao P, Wang L, Wang F. The superior colliculus: cell types, connectivity, and behavior. Neurosci Bull. 2022;38:1519–40. 35484472 10.1007/s12264-022-00858-1 PMC9723059

[45] Knopman DS, Amieva H, Petersen RC, Chételat G, Holtzman DM, Hyman BT, et al. Alzheimer disease. Nat Rev Dis Prim. 2021;7:33. 33986301 10.1038/s41572-021-00269-y PMC8574196

[46] Liu D, Lu J, Wei L, Yao M, Yang H, Lv P, et al. Olfactory deficit: a potential functional marker across the Alzheimer’s disease continuum. Front Neurosci. 2024;18:1309482. 38435057 10.3389/fnins.2024.1309482 PMC10907997

