# Supplementary material for: AAV9-mediated transduction of memory circuits following convection-enhanced delivery into the olfactory bulbs
Source: Gene Ther. 2025 Jul 26;32(6):657–63. doi: 10.1038/s41434-025-00555-4 (PMC12714586; doi:10.1038/s41434-025-00555-4)
Supplement: Supplementary file 1 — Supplementary Fig.1. [file 41434_2025_555_MOESM1_ESM.docx]

**AAV9-mediated transduction of memory circuits following convection-enhanced delivery into the olfactory bulbs.**

Theodore Dimitrov,^1,2,3*^ Vikas Munjal,^1,2,3*^ Allison O’Brien,^1,2^ Matthew Rocco,^1,2^ Ahmad Karkhah,^1,2^ Kaya Ceyhan,^1,2^ Daniel Prevedello,^1^ Lluis Samaranch^1,2^


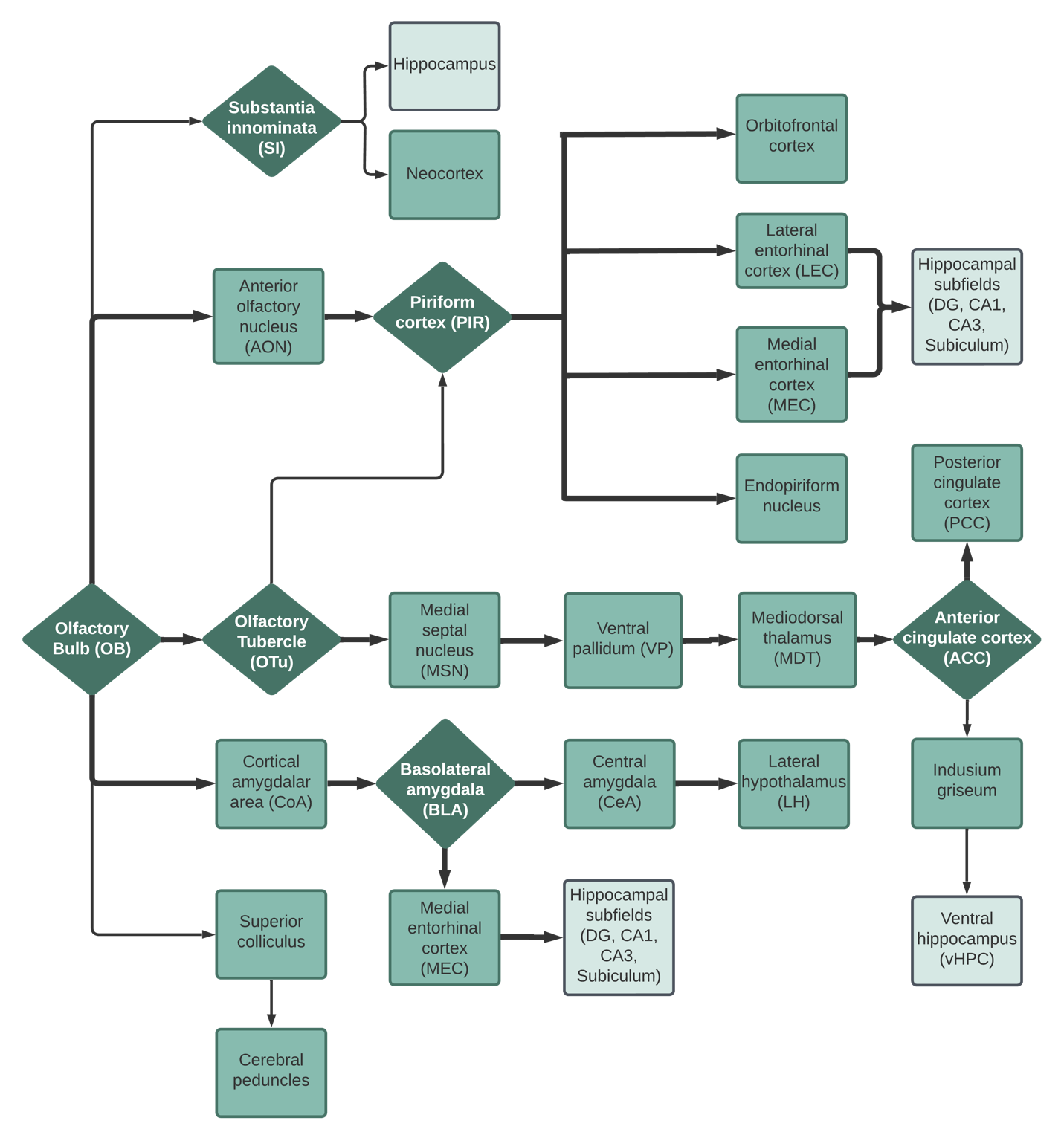


**Supplementary Fig.1.** Simplified projection maps of the regional connectivity between the olfactory bulb (OB), hippocampus (HPC), and key targets. The three main pathways shown are in **bold**: OB-PIR-HPC (upper), OB-OTu-ACC (middle), and OB-CoA-BLA (lower).
